# Preclinical evaluation of the safety and pathogenicity of a live attenuated recombinant influenza A/H7N9 seed strain and corresponding MF59-adjuvanted split vaccine

**DOI:** 10.18632/oncotarget.12746

**Published:** 2016-10-19

**Authors:** Huilin Ou, Wei Yao, Nanping Wu, Frederick X.C. Wang, Tianhao Weng, Chengcong Han, Xiangyun Lu, Dongshan Yu, Haibo Wu, Linfang Cheng, Honglin Chen, Hangping Yao, Lanjuan Li

**Affiliations:** ^1^ State Key Laboratory for Diagnosis and Treatment of Infectious Diseases, Collaborative Innovation Center for Diagnosis and Treatment of Infectious Diseases, The First Affiliated Hospital, School of Medicine, Zhejiang University, Hangzhou, China; ^2^ Department of Pre-clinical Research and Development, Zhejiang Tianyuan Bio-Pharmaceutical Co., Ltd., Hangzhou, China; ^3^ Department of Bioengineering, Erik Jonsson School of Engineering and Computer Science, The University of Texas at Dallas, Texas, USA; ^4^ State Key Laboratory for Emerging Infectious Diseases, Carol Yu Centre for Infection, The University of Hong Kong, Hong Kong, China

**Keywords:** H7N9, safety and toxicity, H7N9 vaccine, H7N9 seed strain

## Abstract

Developing a safe and effective H7N9 influenza vaccine was initiated in early spring 2013, following human infections with a novel avian influenza A (H7N9) virus. In this study, a candidate H7N9 vaccine seed strain is produced using reverse genetics, with HA and NA derived from a human H7N9 virus and the remaining genes from the PR8 backbone virus which grows well in eggs. We verified that the virulence and transmissibility of the recombinant H7N9 vaccine seed strain were decreased as compared to wild-type H7N9 virus, to levels comparable with PR8. Using the seed virus, we produced a monovalent split influenza A (H7N9) MF59-adjuvanted vaccine that was immunogenic in mice. Our H7N9 vaccine is selected for clinical investigation and potential human use. To assess the safety of our H7N9 vaccine, we performed acute toxicity, repeated dose toxicity and active systemic anaphylaxis tests. Our results showed that, under the conditions used in this study, the NOEAL (no obvious adverse effect level) was 30 μg/0.5 mL.

## INTRODUCTION

The outbreak of a previously unrecognized novel reassortant avian influenza A (H7N9) virus in China in March 2013 provoke people's attention [1], the outbreak rapidly spread to other Chinese provinces and municipalities [2]. World Health Organization has reported a total of 571 cases, 212 deaths (37.1%) by February 23, 2015 [3].

Antiviral drugs are critical tools for treating infection with the novel H7N9 virus; however, this approach is fraught with budgetary restrictions and concerns regarding misuse and the development of drug resistance. A more feasible requires the production of safe and effective vaccines [4]. The ideal influenza vaccine seed virus must be low pathogenic for safe use in a manufacturing setting. The application of plasmid-based reverse genetics systems did aid the development and production of vaccines greatly [5, 6], it offers the benefits including the removal of pathogenic traits at the plasmid stage [7]. We applied reverse genetics technologies to construct candidate viruses that harbor the hemagglutinin (HA) and neuraminidase (NA) glycoproteins of A/Zhejiang/DTID-ZJU01/2013(H7N9), with the remaining genes derived from A/Puerto Rico/8/34(H1N1; PR8). There are several H7N9 seed strains, most of them were produced using the HA and NA sequences from the A/Shanghai/2/2013 and A/Anhui/1/2013 strains [8]. Importantly, The A/Zhejiang/1/2013 strain applied in our vaccine was used for the first time. Accordingly, our research was conducted to evaluate recombinant clones in BALB/c mice and ferrets to determine whether the recombinants displayed desirable characteristics including lower virulence and transmissibility. The development of vaccines against H7-containing subtypes have been hampered on account of the poor immunogenicity [9–10]. The oil-in-water adjuvant MF59, which we have paired with our split virus vaccine, has been reported to enhance efficacy, permit dose sparing, lower the antigen dose and improve the antibody response that required to induce protection [11, 12]. In contrast, adding classic alum to subunit influenza or split vaccines has only been reported to improve it to a small extent [13, 14].

The safety profiles of vaccines continue to undergo considerable scrutiny, with a variety of issues raised following the withdrawal of a tetravalent rhesus-human reassortant rotavirus vaccine, RRV-TV, in 1999 [15]. Moreover, vaccines are typically given to a large number of both healthy individuals and vulnerable populations, including infants and young children [16]. Vaccines fall within the scope of medicinal products, and as such must undergo strict preclinical safety evaluation prior to licensing. This MF59-adjuvanted split H7N9 vaccine prepared from recombinant virus has been proven immunogenic and has shown prophylactic efficacy against A/Zhejiang H7N9 virus in preclinical studies [17]. Here, preclinical safety testing including acute toxicity, repeated dose toxicity and active systemic anaphylaxis tests were performed on our vaccine to assess the safety of the vaccine for potential clinical application.

## RESULTS

### Virulence and transmissibility of vaccine seed virus

The pathogenicity of different viruses was compared using the quantitative estimation minimal lethal dose (MLD50). The laboratory strain PR8, caused morbidity and mortality in infected mice, with a MLD50 of 10^-3.5^/0.05 mL. The wt H7N9 virus was highly pathogenic in mice (MLD50, 10^-0.7^/0.05 mL), whereas the pathogenicity of recombinant H7N9 vaccine seed was attenuated (MLD50, 10^-1.6^/0.05 mL), compared with wt H7N9 virus.

No ferret deaths occurred in any of the six groups. In both the wt H7N9 and PR8 virus infection groups, we observed reduced locomotor activity and typical viral upper respiratory symptoms such as sneezing and runny nose beginning around 3 dpi and progressing until 8 dpi. Respiratory symptoms were observed in one ferret in the H7N9 vaccine seed virus infection group at 7 dpi, but disappeared within 24 h. One and two ferrets in the wt H7N9 virus transmission group were found to have clinical symptoms at day 6 and day 7, respectively, after housing with infected ferrets, with all clinical symptoms disappearing by day 10. No obvious clinical symptoms were observed in the other two transmission groups. These observations support that the wt H7N9 virus can transmit by aerosol, causing mild symptoms in ferrets, while the recombinant H7N9 vaccine seed virus does not.

**Figure 1:**
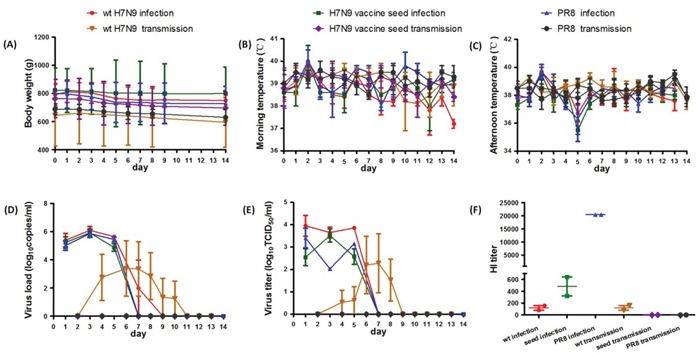
Body weight, body temperature, viral load, viral titers and serum HI titer of ferrets from the virulence and transmissibility test **A.** Weight of ferrets up to 14 dpi. **B.** Morning temperature of the six groups over the course of infection. **C.** Afternoon temperatures of the six groups. **D.** Viral loads in the six groups. Turbinate swabs were collected and viral load determined by qRT-PCR. The geometric mean of the titers is displayed. **E.** Virus titers in the six groups. The titers in the swabs were determined by end-point titration in MDCK cells. **F.** HI titers in serum 14 dpi determined by HI test.

We weighed the three infection groups before infection and again at 2, 4, 6, 8, 10, 12 and 14 dpi, and the three transmission groups before caging together with the infection groups and again on days 2, 4, 6, 8, 10, 12 and 14 after caging together. Total body weight throughout the course of infection (14 days) for each group is shown in Figure 1A. A trend towards a reduction in weight at 1 dpi was observed in all three infection groups and continued until day 8 dpi before reaching a plateau. There was no significant decrease in body weights in the H7N9 vaccine seed virus transmission group or PR8 transmission group. Ferrets in the wt H7N9 virus transmission group started losing weight at day 2 after caging together, with weights remaining unchanged at days 4, 6 and 8, and then increasing. No significant differences in body weight (p > 0.05) were observed across the three infection groups or the three transmission groups.

Body temperature was assessed twice daily and found to fluctuate between the morning and the evening (Figure 1B and 1C). The morning temperatures of the three infection groups increased at 2, 6, and 10 dpi. Both morning and afternoon temperatures peaked at 2 dpi. The morning temperatures of the three transmission groups fluctuated upwards at day 1 and persisted until day 6 after caging together. Body temperatures of the H7N9 vaccine seed virus and PR8 transmission groups fluctuated within the physiological temperature range with no significant differences observed. The temperatures of the wt H7N9 virus transmission group increased at day 2 and day 6 after caging together with mean temperatures of 39.3 ± 0.1°C and 39.2 ± 0.4°C, respectively.

In the wt H7N9 infection group, a viral load of 4.71 log10 ~ 6.65 log10 copies/mL was detected from nasal turbinate swabs at 1~5 dpi by qRT-PCR (Figure 1D). One ferret continued to shed virus until 7 dpi. The period of virus shedding for the H7N9 vaccine seed infection group and the PR8 infection group was similar, up to 5 dpi, with viral loads of 4.32 log10 ~ 6.60 log10 copies/mL and 4.55 log10 ~ 6.39 log10 copies/mL respectively. The viral load of the H7N9 vaccine seed infection group was significantly lower than the wt H7N9 virus infection group (p < 0.05) and PR8 infection group (p < 0.05). No virus was detected from the nasal turbinate swabs of the H7N9 vaccine seed transmission group or PR8 transmission group. Two ferrets from the wt H7N9 transmission group showed positive viral loads (4.51 log10 ~ 7.28 log10 copies/mL) from 4 to 8 dpi. One of the ferrets had a detectable viral load for 7 days.

Virus titers from the H7N9 vaccine seed infection group were significantly lower than the wt H7N9 virus infection group (p < 0.05; Figure 1E), especially at 1 and 5 dpi. Titers were also lower than in the PR8 infection group (p < 0.05), especially at 3 dpi. The peak of viral shedding from ferrets in the transmission groups was between 5 and 8 dpi, which was consistent with the timeframe of virus shedding from ferrets in the infection groups. The virus titers from nasal turbinate swabs of the wt H7N9 transmission group were even higher than from the wt H7N9 infection group at certain times during observation period; the peak virus titer from the wt H7N9 virus transmission group was 4.92 log10 TCID50/mL, while that of the wt H7N9 virus infection group was 4.30 log10 TCID50/mL. The virus titer of the H7N9 virus seed transmission group was significantly lower than the other two transmission groups (both p < 0.05).

The serum HI titers of all the groups were negative at 3 dpi and 14 dpi (Figure 1F). The serum HI titers varied across the three infection groups with the PR8 infection group showing the highest titers (20480), followed by the H7N9 seed infection group (453), and finally the wt H7N9 infection group (113). Fourteen days after caging together, the HI titers of 2 ferrets in the wt H7N9 transmission group were 80 and 160 respectively, which suggests these two ferrets may have been infected through aerosol transmission.

**Figure 2:**
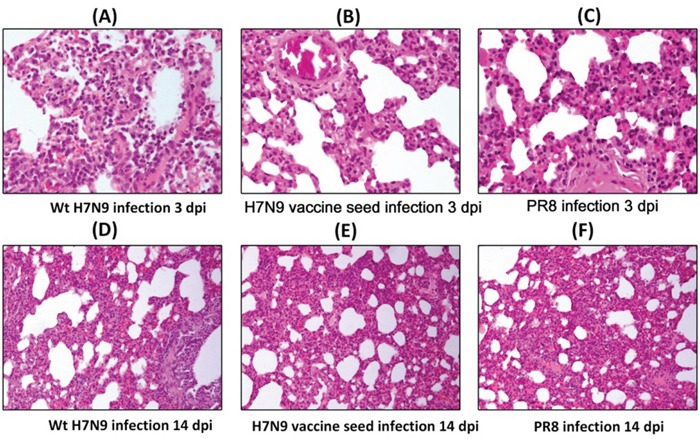
Lung histopathology of ferrets inoculated intranasally inoculated with 10^6^ TCID50 of wt H7N9 virus, H7N9 vaccine seed or PR8 at 3 dpi and 14 dpi Multiple 4-μm-thick sections were stained with H&E. Original magnification **A-C.** ×200, **D-F.** ×100.

The extent and characteristics of the lesions were variable among the three infection groups (Figure 2). Pulmonary vasodilation occurred in all three infection groups at 3 dpi and more serious damage such as focal widened alveolar septum, epithelial cell degeneration and shedding and inflammatory cell infiltration were detected in wt H7N9 virus infected ferrets. Lesions were mitigated in all these groups at 14 dpi. The wt H7N9 infected ferrets showed the most severe lesions at both 3 dpi and 14 dpi. No inflammatory reactions or other histopathological changes were observed in any of the following tissues: spleen, intestines, liver, heart, kidney, olfactory bulb and brain.

### Toxicity of the H7N9 split vaccine

**Figure 3:**
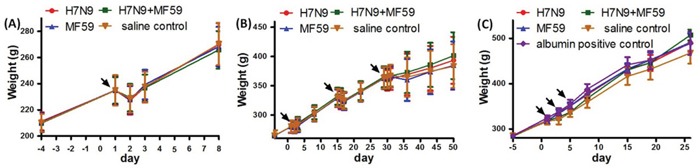
Weight change of rats from the A. acute toxicity test, B. repeated dose toxicity test, and C. active systemic anaphylaxis test The arrows indicate the immunization times.

There were no unscheduled deaths during the course of the study. No vaccine-related effects on clinical signs, body weight (Figure 3), food consumption, ophthalmologic examinations, coagulation, urinalysis, body temperature or local tolerance were observed.

Acute toxicity studies showed that NEUT and WBC in both males and females from group 3 (H7N9 split virus with MF59 adjuvant) and group 4 (MF59 adjuvant control) increased, with values being comparable to the NS (normal saline) control group (group 1; Table 1A). The albumin/globulin (A/G) ratio in both males and females in group 3 and group 4 decreased (Table 1B). There were no H7N9 avian influenza vaccine-related organ weight changes, H7N9 avian influenza vaccine with adjuvant, or adjuvant-related organ weight changes observed in the spleen. Compared with the NS control group, the mean absolute and relative (to brain and body weight) weight of the spleen of females in the group treated with adjuvant increased slightly (22% to 24%; Table 1C), without gross lesions. All spleen weights for other animals were within the range of the NS control groups with the exception one male animal in group 4 which had a higher spleen weight. Similar changes did not occur in other males; therefore, it was considered unrelated to the study treatments.

**Table 1:** Hematology: NEUT and WBC; Serum Chemistry: ALB (Albumin), GLB (Globulin) and A/G (%) and Spleen weight (absolute weight, spleen/body ratio and spleen/brain ratio) in both males and females rats from acute toxicity test in group 2 (H7N9 split virion), group 3 (H7N9 split virion with MF59 adjuvant) and group 4 (MF59 adjuvant control) with values being comparable to the NS control group (Group 1)

Table 1A							
Dose (mg/kg)	Study Day	30 μg/0.5 mL H7N9 Male:10/5, Female:10/5		15 μg/0.25 mL H7N9 + MF59 0.25 mL Male:10/5, Female:10/5		0.25 mL PBS + MF59 0.25 mL Male:10/5, Female:10/5	
Number of animals							
NEUT (10^3^/μL) (%)	Day 2	↓0.5	↑12.65	↑402.84***	↑706.08***	↑366.95***	↑765.21***
	Day 14	↓36.75	↑3.32	↑4.3	↑42.71	↑0.72	↑14.57
WBC (10^3^/μL) (%)	Day 2	↓14.68	↑7.84	↑26.25	↑61.33**	↑17.69	↑77.25**
	Day 14	↓40.43**	↑7.67	↓19.38	↑0.87	↓26.79*	↑18.02

Table 1B							
Dose (mg/kg)	Study Day	30 μg/0.5 mL H7N9 Male:10/5, Female:10/5		15 μg/0.25 mL H7N9 + MF59 0.25 mL Male:10/5, Female:10/5		0.25 mL PBS + MF59 0.25 mL Male:10/5, Female:10/5	
Number of animals							
Day 2	ALB (g/L)	↓2	−	↓1.9	↓0.8	↓2	↓2.1
	GLB (g/L)	↓1.6	↓0.2	↑1.7	↑2.1	↑1.8	↑2.3
	A/G (%)	↑0.62	↑0.88	↓11.11***	↓9.34**	↓12.03***	↓12.9***
Day 14	ALB (g/L)	↓1.1	↓1.3	↓.1.8	↓3.5	↓0.6	↓2.2
	GLB (g/L)	↓0.8	↑1.6	↓0.9	↑0.6	↓0.6	↑1.8
	A/G (%)	↑0.24	↓2.71	↓1.72	↓4.02	↑0.8	↓3.77

Table 1C							
Dose (mg/kg)	Index	30 μg/0.5 mL H7N9 Male:10/5, Female:10/5		15 μg/0.25 mL H7N9 + MF59 0.25 mL Male:10/5, Female:10/5		0.25 mL PBS + MF59 0.25 mL Male:10/5, Female:10/5	
Number of animals							
Spleen weight	Spleen (g)	↓0.0231	↑0.0227	↓0.0415	↑0.0245	↓0.0688	↑0.1106
	Spleen/Body (10^-3^)	↓0.0033	↑0.0476	↓0.0805	↑0.0506	↓0.2374	↑0.4543
	Spleen/Brain	↓0.0008	↑0.002	↓0.0093	−	↓0.0119	↑0.0537

“−” indicates that all changes were small in magnitude or were comparable to the control groups. ***, ** and* indicate p < 0.001, 0.01, and 0.05, respectively when compared with the NS control. (A) NEUT and WBC changes after dosing on day 2 (B) Serum ALB, GLB and A/G (%) changes of after dosing on day 2 (C) Spleen weight (absolute weight, spleen/body ratio and spleen/brain ratio) changes after dosing on Day 14.

NEUT and EOS in both males and females undergoing repeated dose toxicity testing in group 3 (H7N9 split virus with MF59 adjuvant) and group 4 (MF59 adjuvant control) increased after dosing on day 17 and day 32 (Table 2A). A/G ratio in both males and females in group 3 and group 4 decreased after dosing on day 17 and day 32, when compared to the NS control (Table 2B). The ratio change was due to a significant increase in globulin, and decrease in albumin. In the dosing phase, mean absolute and relative (to body and brain) spleen weights were increased in males and females treated with the influenza vaccine with adjuvant and increased to statistically significant levels (p < 0.05) in females treated with adjuvant alone (Table 2C). The weight change of the spleen was associated with minimal extramedullary hematopoiesis in some females. Following the recovery period, the hematology (NEUT and EOS), serum chemistry (A/G ratio) and spleen weight changes had resolved, with values comparable to the NS control group.

**Table 2:** Hematology: NEUT and EOS; Serum Chemistry: ALB (Albumin), GLB (Globulin) and A/G (%) and spleen weight (absolute weight, spleen/body ratio and spleen/brain ratio) in both male and female rats from repeated dose toxicity testing in group 2 (H7N9 split virion), group 3 (H7N9 split virion with MF59 adjuvant) and group 4 (MF59 adjuvant control) with values being comparable to the NS control group (group 1)

Table 2A							
Dose (mg/kg)	Study Day	30 μg/0.5 mL H7N9 Male:10/5, Female:10/5		15 μg/0.25 mL H7N9 + MF59 0.25 mL Male:10/5, Female:10/5		0.25 mL PBS + MF59 0.25 mL Male:10/5, Female:10/5	
Number of animals							
NEUT (10^3^/μL) (%)	Day 17	−^a^	−	↑50.14***	↑143.29***	↑54.96***	↑75.58***
	Day 32	−	−	↑44.83	↑66.75*	↑7.92	↓0.48
	Day 57	−	−	↑2.83	↓32.56	↑29.09	↓28.37
EOS (10^3^/μL) (%)	Day 17	−	−	↑149.73***	↑227.27***	↑98.46***	↑145.46***
	Day 32	−	−	↑366.67***	↑613.04***	↑69.44**	↑215.22***
	Day 57	−	−	↑40	↑83.33	↑20	↑8.33

Table 2B							
Dose (mg/kg)	Study Day	30 μg/0.5 mL H7N9 Male:10/5, Female:10/5		15 μg/0.25 mL H7N9 + MF59 0.25 mL Male:10/5, Female:10/5		0.25 mL PBS + MF59 0.25 mL Male:10/5, Female:10/5	
Number of animals							
Day 17	ALB (g/L)	↑0.1	↓2.1	↓3.2***	↓5***	↓2.4***	↓4.5***
	GLB (g/L)	↓0.8	↓1	↑3***	↑2.4***	↑2.6***	↑1.3
	A/G (%)	−	−	↓17.05***	↓18.36***	↓14.06***	↓14.07***
Day 32	ALB (g/L)	↓0.4	↓0.8	↓2.2***	↓4**	↓1.8***	↓3.1*
	GLB (g/L)	↓0.6	↑0.3	↑3.7***	↑4.8***	↑1.6*	↑1
	A/G (%)	−	−	↓18.60***	↓24.56***	↓10.72***	↓11.53***
Day 57	ALB (g/L)	↑0.3	↓1.7	↑0.3	↓1.3	↓0.4	↑2.3
	GLB (g/L)	↓0.7	↓0.4	↑2.5	↑3.3	↓1.2	↑1.1
	A/G (%)	−	−	↓8.06	↓11.57*	↑3.70	↑2.57

Table 2C							
Dose (mg/kg)	Index	30 μg/0.5 mL H7N9 Male:10/5, Female:10/5		15 μg/0.25 mL H7N9 + MF59 0.25 mL Male:10/5, Female:10/5		0.25 mL PBS + MF59 0.25 mL Male:10/5, Female:10/5	
Number of animals							
Day 32	Spleen (g)	↑0.0638	↑0.0201	↑0.1103	↑0.0725	↑0.0399	↑0.0849*
	Spleen/Body (10^-3^)	↑0.1304	↑0.0617	↑0.2348	↑0.2504	↑0.0343	↑0.3262*
	Spleen/Brain	↑0.0203	↑0.0203	↑0.0421	↑0.0421*	↑0.0194	↑0.0399*
Day 57	Spleen (g)	↓0.0019	↑0.1035	↓0.0755	↓0.0079	↓0.0421	↓0.0102
	Spleen/Body (10^-3^)	↓0.0138	↑0.0175	↓0.0978	↑0.0646	↓0.0082	↓0.0195
	Spleen/Brain	↓0.0138	↑0.0152	↓0.0329	↑0.0052	↓0.0189	↑0.0033

“−” means that all changes were small in magnitude or were comparable with the control groups. ***, **and* indicate p < 0.001, 0.01, and 0.05, respectively when compared with the NS control. (A) NEUT and EOS changes after dosing on Day 17, 32 and 57. (B) Serum ALB, GLB and A/G (%) changes after dosing on Day 17, 32 and 57. (C) Spleen weight (absolute weight, spleen/body ratio and spleen/brain ratio) changes after dosing on Day 32 and 57.

**Figure 4:**
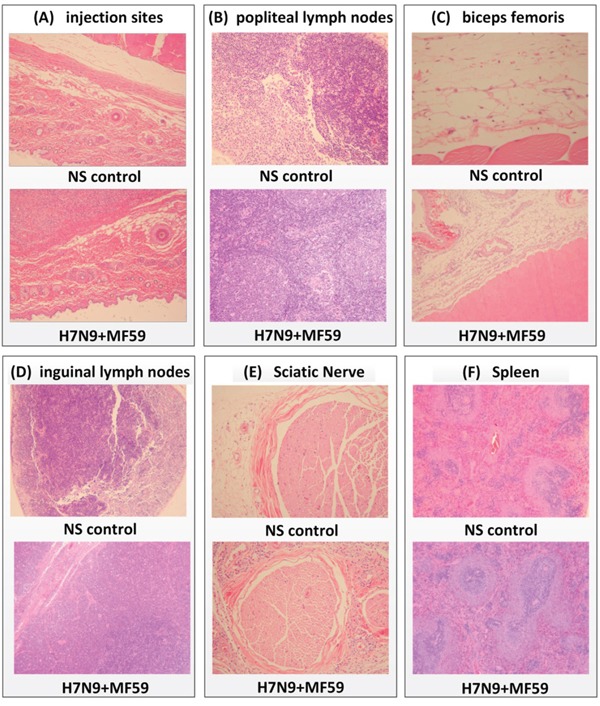
At day 32 (dosing phase) of the repeated dose toxicity test, H&E staining was performed of various organs from rats treated with H7N9 vaccine with MF59 adjuvant or NS control **A.** Intramuscular injection sites (original magnification, ×40), **B.** popliteal lymph nodes (original magnification, ×200), **C.** biceps femori (original magnification, NS control, ×40, H7N9 + MF59, ×200), **D.** inguinal lymph nodes (original magnification, ×40), **E.** sciatic nerve (original magnification, ×100), **F.** spleen (original magnification, ×40).

**Figure 5:**
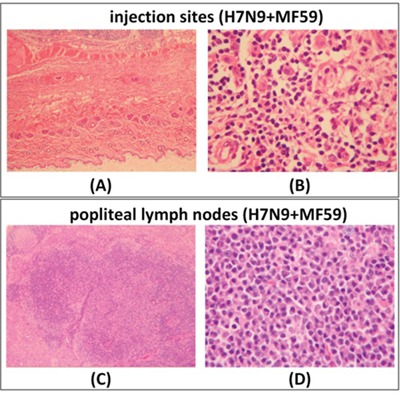
At day 57 (recovery phase) of the repeated dose toxicity test, H&E staining was performed of intramuscular injection sites and popliteal lymph nodes from rats treated with H7N9 vaccine with MF59 adjuvant Intramuscular injection sites, magnification ×40 **A.**, ×400 **B.** Popliteal lymph nodes magnification ×40 **C.**, ×200 **D.**

At the dosing phase of repeated dose toxicity testing, treatment with the influenza vaccine with adjuvant, and/or treatment with adjuvant alone, caused mixed cell infiltration in the interstitium of the intramuscular injection sites (Figure 4A). The severity of this change was slightly higher in animals treated with the influenza vaccine with adjuvant than in those treated with adjuvant alone. We also observed lymphoid hyperplasia in the follicles and plasmacytosis in the medulla of the lymph nodes (popliteal and inguinal; Figure 4B and Figure 4D). These lymph nodes were the draining nodes for the injection and these changes were an expected effect of the treatment. Mixed cell infiltration in the perinodular connective tissue of the popliteal lymph nodes, in the interstitium of the skeletal muscle (biceps femoris, Figure 4C), and in the epineurium surrounding the nerves (Figure 4E) was observed. These findings were considered an extension of the local injection reaction because of the close proximity to the intramuscular injection site(s). Extramedullary hematopoiesis in the spleen (Figure 4F) was also observed, which correlated with increased organ weights. This change was considered to be an adaptive response to the inflammatory reaction incited by the injection of adjuvant or influenza vaccine with adjuvant, which correlated with the NEUT and EOS increase in hematology analysis and A/G decrease in serum chemistry. At the end of recovery phase (Figure 5), only mononuclear cell infiltration and fibroplasias at the injection sites, and lymphoid hyperplasia and plasmacytosis in the popliteal lymph nodes were noted, indicating most of the changes had resolved following the recovery phase, which correlated with the recovery observed in the hematology and serum chemistry results.

No abnormalities were noted during the induction phase of the active systemic anaphylaxis test. On the days of challenge dosing, no abnormalities were recorded for groups 1-4 immediately following the challenge injection, up to 3 h after injection. For animals in group 5 (albumin, positive control), strong signs of labored respiration, unsteady gait and/or purpura, as well as weak signs of restlessness, piloerection, and nose scratching, were observed in six animals within 5 min of intravenous challenge.

Hematology and clinical pathology changes were restricted to those animals receiving the vaccine with adjuvant or adjuvant alone. These included significant increases in WBC and NEUT in the acute toxicity test, increases in EOS and NEUT in repeated dose toxicity testing and decreases in the A/G ratio. These changes were considered an immune response to the adjuvant. There were no macroscopic findings in the observation period. There were no organ weight changes or microscopic findings that were attributed to administration of the influenza vaccine alone. During repeated dose toxicity testing, increased mean absolute and relative spleen weights were observed in males and females treated with influenza vaccine with adjuvant and statistically significant increases were observed in females treated with adjuvant alone. This change correlated with minimal extramedullary hematopoiesis in some females, and was considered to be an adaptive response to the inflammatory reaction incited by the injection of influenza vaccine with adjuvant or adjuvant alone. Consequently, under the conditions in this study, the NOEAL (no obvious adverse effect level) was determined to be 30 μg/0.5 mL.

## DISCUSSION

Previous studies have reported the safety of vaccines prepared using alternative seed strains [18]. However, most of the studies are clinical reports and evaluate only the clinical adverse events. Systematic reports including potential physiological and pathological changes are rare. Pathogenicity experiments in mice and ferrets confirmed that we successfully developed a safe vaccine seed strain which demonstrated lower virulence and transmissibility as compared with wt H7N9 virus. Furthermore, preclinical safety and toxicity tests of the MF59-adjuvanted split influenza vaccine provided no indication of a potential risk of administering the vaccine up to 30 μg/0.5 mL dose in the experimental animals.

We applied reverse genetics to construct candidate vaccine which contained the HA and NA genes of A/Zhejiang/DTID-ZJU01/2013 virus and six genes that encoded internal proteins from the PR8 virus. According to a previous analysis of antigenic epitopes of avian influenza A (H7N9) viruses [19, 20], the antigenic variation of HA fragments between A/Zhejiang H7N9 virus and other H7N9 viruses is minor. The H7N9/PR8 virus has many desirable characteristics, including that it can be parenterally administered in an inactive form. Moreover, the lack of pathogenicity or infectivity in chickens should provide for the production of an egg-based vaccine. We utilized a ferret (*Mustela putorius furo*) model to investigate virulence and transmissibility of this H7N9 vaccine seed strain because they are susceptible to natural infection and develop respiratory disease and lung pathology similar to humans [21]. Pathogenicity studies showed that the H7N9/PR8 reassortant was not highly virulent, and transmissibility for mice or ferrets, which was in accordance with ideal influenza vaccine seed strain requirements.

Unfortunately, an adjuvant is demanded because of the comparatively week immunogenicity of split vaccines [9, 22]. MF59 was applied because according to the report that it can obviously reduce the antigen requirements of the split influenza vaccine and can achieve the protective effect with a single dose [10, 23]. Ideal adjuvant properties include a strong enhancement of the immune response, as well as a favorable safety profile. Similar to the classic immunoadjuvant Al(OH)_3_, the safety and potency of MF59 has been described in many human clinical trials [24]. The safety of vaccines must be strictly assessed, and every vaccine batch must be examined by the National Control Laboratories according to the guidelines published by each country. However, rare cases of the development of autoimmune diseases following influenza vaccinations with MF59 adjuvant [25] and alum-associated macrophagic myofasciitis syndrome [26] have been reported. The preclinical study reported here supports the good safety of the MF59-adjuvanted H7N9 split vaccine by suggesting a lack of toxicological findings. Toxicity tests of the split H7N9 vaccine in rats and guinea pigs indicated a large safety margin. A dose of a high concentration of the MF59-adjuvanted H7N9 split vaccine by the intramuscular route in rats showed no effects at levels far above a typical clinical dose volume of 0.5 mL for these types of materials. Therefore, on a weight-to-weight basis, a safety margin of approximately 240-fold (rat = 250 g) was achieved for a 60 kg person. Only after repeated intramuscular administration of the vaccine with MF59 or MF59 alone in rats did we observe changes in hematology and clinical pathology, and these changes were considered an immune response to the adjuvant or adaptive response to the inflammatory reaction. Overall, the vaccine candidate showed no toxicological findings of significance at levels greatly in excess of those proposed for clinical use. However, the causes of clinical adverse events are often unclear, and careful attention should be paid especially during the use of concomitant medications.

In conclusion, the comprehensive preclinical evaluation program conducted here documented the safety of the recombinant H7N9 vaccine seed strain and safety of the intramuscular MF59-adjuvanted split influenza vaccine in animal models. We believe this MF59-adjuvanted H7N9 split vaccine is able to protect against H7N9 virus without major HA antigenic drift. Further human testing will be needed to determine whether it could represent a candidate vaccine.

## MATERIALS AND METHODS

### Ethics statement

Research has been carried out in accordance with the ethical standards, and according to the Declaration of Helsinki and national and international guidelines. The investigation has been approved by the authors' institutional review board.

### Animals, adjuvant and cell lines

Six-week-old BALB/c mice and 4- to 6-month-old castrated male pet ferrets (*Mustela putorius furo*) were purchased from the Institute of Laboratory Animal Science, Chinese Academy of Medical Sciences. Six-week-old rats were supplied by BioLASCO Taiwan Co., Ltd. Four- to six-week-old guinea pigs were provided by Charles River (Beijing, China). We housed animals in the departmental animal facility under the following requirements: temperature 22 ± 3°C, humidity 65 ± 5%, and a 12 h light/dark cycle. The animals were fed a standard diet and water ad libitum, and were acclimatized for one week prior to the experiments. MF59 adjuvant was kindly provided by Tianyuan Bio-Pharmaceutical, which is affiliated with Novartis Vaccine Inc. The non-permissive renal canine epithelial cell line MDCK was obtained from ATCC (Rockville, MD, USA). All the tests performed conformed to Good Laboratory Practice (GLP).

### Virus preparation

When using live influenza virus, we performed it in a biosafety level (BSL) 3 laboratory according to standard BSL3 guidelines. The reassortant virus was constructed using plasmid-based reverse genetics through transfection of eight individual plasmids into Vero cells [5, 6]. The plasmids harbored the HA and NA genes from the A/Zhejiang/DTID-ZJU01/2013(H7N9) virus, which was isolated from a patient in Zhejiang Province of China in 2013, and six internal genes from PR8. These viruses were propagated in specific-pathogen-free (SPF), embryonated chicken eggs and harvested, clarified, ultrafiltrated, zonal centrifuged, purified, cleaved, sterilization filtered and identified. All production processes were completed in a sterile workshop at Tianyuan Bio-Pharmaceutical in accordance the requirements of China Food and Drug Administration (CFDA) standard.

### Vaccine seed virus MLD50 determination

Titrations of wild-type (wt) H7N9 virus (A/Zhejiang/DTID-ZJU01/2013), the H7N9 vaccine seed (A/ZJU01/PR8/2013) and a PR8 virus control were performed by intranasal inoculation of 13 to 14 g BALB/c mice with 50 μL of virus at 10-fold serial dilutions. MLD50 in mice was determined in groups of six mice. The MLD50 was calculated using the Reed and Muench method [27].

### Virulence and transmissibility of the vaccine seed virus

The wt H7N9 virus, H7N9 vaccine seed virus and PR8 virus control were tested for virulence and transmissibility in total of 24 (350 g to 900 g) ferrets. The ferrets were divided into six groups for infection testing (wt H7N9 virus infection, H7N9 vaccine seed virus infection and PR8 virus infection), or transmission testing (wt H7N9 virus transmission, H7N9 vaccine seed virus transmission and PR8 virus transmission). Ferrets were housed individually. Four ferrets per infection group were inoculated with 10^6^ TCID50 of virus. On day 1 post-infection (dpi), a naïve ferret was introduced into each cage with the inoculated ferrets to measure direct contact transmission. All animals were observed for clinical signs of respiratory disease twice daily. Body weights were recorded and turbinate swabs collected every other day. Ferrets in the three infection groups were sacrificed (two at each time point) at 3 dpi and 14 dpi and lungs were removed, fixed in 10% buffered formalin, and embedded in paraffin. Blood samples were collected just prior to sacrifice, and serum samples were tested for hemagglutination inhibition (HI) titers. Viral load and viral titers in the lung (each lobe), turbinate, olfactory bulb, brain (anterior and posterior), spleen and duodenum were determined by quantitative real-time PCR and end-point titration in MDCK cells respectively. Lung (each lobe), spleen, intestines, liver, heart, kidney, olfactory bulb, brain (anterior and posterior), and other organs were reserved for pathological examination. In each transmission group, the period of infection, viral load and viral titers were determined in the same manner as described above. HI titers were examined at 14 days post-conversion.

### HI test

All sera were processed using receptor-destroying enzyme to prevent non-specific inhibition, as described previously [11]. Prior to testing, 4 hemagglutinating units of virus was mixed 1:1 with a two-fold serial dilution series of serum and incubated at 37°C for 1 h. Following the incubation, 50 μL of 1% chicken erythrocytes in phosphate buffered saline (PBS) were added, mixed, incubated for 1 h at 4°C, and agglutination patterns read within 10 min. All tests were performed in “V” bottom microtiter plates and carried out in duplicate. The HI-titer was defined as the reciprocal of the last dilution of serum that completely inhibits hemagglutination.

### RT-PCR

The nasal turbinates were gently rubbed with a wet cotton swab tip for 5 seconds to collect the swab samples. Before extraction, 0.5 mL Hank's Balanced Salt Solution (HBSS) was added into the secretions in NS to adjust to 1 mL volume. Samples were extracted from 200 μL as previously described [28]. Quantitative RT-PCR using Influenza A H7N9 virus nucleic acid fluorescent PCR rapid detection kits (DaAn Gene) was performed to amplify the HA and NA genes of the H7N9 virus, with 18S rRNA serving as an internal control.

### Virus titrations

We determined virus titrations as described by end-point titration in MDCK cells [29]. MDCK cells were inoculated with tenfold serial dilutions of culture supernatants. Cells were washed twice with PBS one hour after inoculation and cultured in 200 μL of infection medium (MEM containing 100 IU/mL penicillin, 100 μg/mL streptomycin, 4% bovine serum albumin [fraction V, Gibco-BRL], and 4 μg/mL trypsin [Gibco-BRL]). Three days after inoculation, the supernatants of cell cultures were tested for agglutinating activity using turkey erythrocytes as an indicator of infection of the cells. Infectious virus titers were calculated from five replicates each of the homogenized tissue samples [27].

### Histopathology of vital organs

All vital organs including the lungs (each lobe), spleen, intestines, liver, heart, kidney, olfactory bulb, and brain (anterior and posterior) from the three infection groups were harvested and immersed in 4% formaldehyde at least for 24 h. After fixation of the tissues and processing in paraffin wax, 4 μm sections were prepared and stained with H&E, examined and photographed.

### Safety and toxicity evaluation of the split virus vaccine and MF59 adjuvant acute toxicity in rats

According to the WHO guidelines on vaccine safety evaluation (WHO technical reports, No. 927, 2005), the recommended dosing volume for each injection is no more than 30 μg/0.5 mL. Therefore, in this study, the highest single dose for the H7N9 avian influenza vaccine was 30 μg/0.5 mL. Forty Sprague Dawley (SD) rats were randomly assigned to four groups of 5/sex/group. Rats from groups 1-4 were intramuscularly injected with split H7N9 vaccine (30 μg/0.5 mL), split H7N9 vaccine (15 μg/0.25 mL) and MF59 0.25 mL, MF59 (PBS 0.25 mL and MF59 0.25 mL), or NS 0.5 mL, respectively. The dose was administered by intramuscular injection, with a half dose infected in each hind limb. Animals were observed for 14 days then fasted overnight prior to being necropsied.

### Repeated dose toxicity in rats

A total of one hundred and twenty SD rats were randomly assigned to four groups of 15/sex/group. Rats from groups 1-4 were intramuscularly injected with split H7N9 vaccine (30 μg/0.5 mL), split H7N9 vaccine (15 μg/0.25 mL) and MF59 0.25 mL, M59 (PBS 0.25 mL and MF59 0.25 mL), or NS 0.5 mL, respectively, three times on days 1, 15, and 29, as described above. Ten rats of each sex per group and 5/sex/group rats underwent exsanguination from the abdominal aorta after isoflurane anesthesia and blood sample collection and were necropsied on day 32 (dosing phase), and day 57 (recovery phase), respectively.

### Active systemic anaphylaxis test of split vaccine in guinea pigs

A total of 30 guinea pigs of both sexes were randomly assigned to five groups of 5/sex/group. For induction, groups 1-5 were intramuscularly injected with of 0.5 mL NS, 0.25 mL MF59 plus 0.25 mL PBS, 30 μg/0.5 mL H7N9 vaccine, 30 μg/0.25 mL H7N9 vaccine plus 0.25 mL MF59 and 5 mg/0.5 mL albumin, respectively, once every other day for a total of three injections (on days 1, 3, and 5). The challenge was performed on days 19 and 26. For the challenge, all animals were intravenously injected with doses twice those of the induction phase.

### Clinical signs

Cage-side observation was conducted daily for the duration of the study period. Viability (morbidity and mortality) checks were performed twice daily. Detailed observations were conducted once pre-test, once prior to dosing on day 1, once at approximately 3 h post-dosing on day 1, and weekly thereafter during the observation period.

### Body weight

Each animal was weighed once pre-test, once on day 1 prior to dosing, once between 24 h and 48 h post-dosing, and once weekly throughout the remaining observation period.

### Food consumption

Daily food consumption was measured once for all animals at day -2 during the pre-dosing period, and once weekly throughout the study from day -1 (except during fasting periods). Food was withdrawn overnight prior to scheduled blood collections for hematological, coagulation and serum biochemical determinations, urine collections, and scheduled necropsies.

### Ophthalmological examination

Eyes were examined via slit lamp and indirect ophthalmoscope, once pre-dose, once halfway through the experimental period and once on the last day. A mydriatic (containing 1.0% tropicamide) was used to dilate the pupils for ophthalmology examination.

### Local tolerance (injection site)

Injection sites had previously been clipped of hair and the injection site identified by indelible ink. The injection site was maintained free of excessive hair during the observation period. The injection site was scored using the Draize scoring system. The injection site was scored prior to each injection and at approximately 24 h and 48 h after injection.

### Body temperature

Body temperatures were measured once prior to day 1 and pre-dose on day 1, once at approximately (± 30 min) 3 h, 6 h, and 24 h post-dose and once prior to scheduled necropsy.

### Hematological analysis

Blood samples for interim hematology, and clinical chemistry evaluation were obtained from the jugular vein of conscious animals. Blood samples for hematology, coagulation, and clinical chemistry were obtained from the abdominal aorta of animals at necropsy as a terminal procedure after isoflurane anesthesia. Five hundred microliters of blood were transfer into a CBC collection tube (K2EDTA, BD, USA) and analyzed using a hematology analyzer (Advia 2120, Seimens, USA). Additionally, 2 mL blood were transferred into a vacutainer (sodium citrate 3.2%, BD) and centrifuged at 3000 rpm for 10 min. The plasma was isolated and used to determine aggregation time using a coagulometer (BE Compact-X, Behnk Elektronik, Germany). The following parameters were anlayzed: HCT (hematocrit), WBC (white blood cell count), HGB (hemoglobin concentration), RBC (red blood cell count), MCV (mean corpuscular volume), MCHC (mean corpuscular hemoglobin concentration), MCH (mean corpuscular hemoglobin), MPV (mean platelet volume), RDW (RBC distribution width), PLT (platelet count), NEUT (neutrophils), EOS (eosinophils), BASO (basophils), MONO (monocytes), LYMP (lymphocytes), retic (reticulocyte count), blood smear for cytology, PT (prothrombin time), FIB (fibrinogen), APTT (activated partial thromboplastin time).

### Blood chemistry

Serum was isolated and used for biochemical analysis using an automatic serum analyzer (Hitachi 7180, Japan). The following parameters were analyzed: ALT (alanine aminotransferase), CRE (creatinine), AST (aspartate aminotransferase), Ca (calcium), TP (total protein), P (phosphorus), ALB (albumin), GLB (globulin), A/G (albumin/globulin ratio), TG (triglycerides), TCHO (total cholesterol), TBILNA (total bilirubin), ALP (alkaline phosphatase), Na (sodium), GGT (gamma glutamyl transferase), K (potassium), GLU (glucose), Cl (chloride), UREA (urea), CK (creatine kinase).

### Urinalysis

During the final week of study, urinalysis was conducted to determine BIL (bilirubin), turbidity (color and clarity), KET (ketones), SG (specific gravity), BLO (occult blood), pH, PRO (protein), GLU (glucose) and URO (urobilinogen) using Multistix 10 SG reagent strips (Bayer, U.S.A.), and a urine analyzer (AX-4030, Aikelai, Japan). Microscopy studies were also performed for examination of sediment.

### Necropsy findings

Scheduled terminated study animals underwent exsanguination from the abdominal aorta after isoflurane anesthesia. Gross observation was performed of the external surface, the cranial cavity, all orifices, and all thoracic and abdominal organs.

### Organ weight

Following necropsy, the absolute and relative weight ratio (organ-to-body and organ-to-brain) of following organs was measured: adrenal glands, brain, heart, liver, kidneys, epididymis, lungs with main stem bronchi, ovaries, pituitary gland, prostate gland, thymus, spleen, testes, thyroid glands with parathyroid gland(s) and uterus (including cervix).

### Histopathology

The following tissues were obtained: liver, kidney, adrenal gland, bone, aorta and bone marrow (sternum), bone (femur, including stifle joint), fallopian tubes, harderian gland, heart, injection sites, lungs with main stem bronchi, brain, spleen, epididymis, uterus, vagina, trachea, nerves (sciatic), esophagus, mammary glands (inguinal, female only), pituitary gland, prostate gland, salivary glands, lymph nodes (inguinal, mandibular, mesenteric and popliteal), thymus, stomach, urinary bladder, small/large intestine, eyes with optic nerve(s), ovaries, seminal vesicles, skin (inguinal), skeletal muscle (biceps femoris), spinal cord (cervical, thoracic, lumbar), testes, thymus, gross lesions, submandibular gland and pancreas. Tissues were trimmed and fixed in 10% neutral buffered formalin (NBF) with the following exceptions: eyes with optic nerves were fixed in 2.5% glutaraldehyde solution, and testes as well as epididymis were fixed in modified Davidson's solution for 24 to 72 h before being transferred to 10% NBF. Tissues were removed from fixative, processed, embedded in paraffin, sectioned, stained with hematoxylin and eosin (H&E), and examined microscopically.

### Statistical analysis

Males and females were analyzed separately. The viral load, virus titer, body weight, organ weight, biochemistry data and hematological data were analyzed for homogeneity of variance using Levene's test. One-way ANOVA was conducted for data shown to have homogeneity of variance. If homogeneity of variance and significance between test groups was confirmed, the Scheffe's test was performed. If heterogeneity of variance and significance between test groups was determined, Dunnett's test was conducted. All analysis were performed using the SPSS program.
